# Abnormal changes in neuropsychological function, brain structure and cerebral perfusion in patients with unruptured intracranial aneurysms

**DOI:** 10.3389/fneur.2024.1463156

**Published:** 2024-10-08

**Authors:** Wei Li, Si Zhang, Weijie Fan, Xiaomei Fu, Dong Zhang, Li Wen

**Affiliations:** Xinqiao Hospital, Chongqing, China

**Keywords:** unruptured intracranial aneurysm, voxel-based morphometry, surface-based morphometry, cerebral blood perfusion, cognitive impairment, emotional disorder

## Abstract

**Background:**

Patients with unruptured intracranial aneurysms (UIAs) often experience emotional changes and cognitive impairments. However, the specific mechanisms underlying these impairments are still not fully understood.

**Methods:**

In the present study, voxel-based morphometry (VBM) and surface-based morphometry (SBM) were employed to investigate structural alterations in 49 patients diagnosed with UIAs compared with 50 healthy controls. Additionally, this study aimed to analyze the correlations among cortical morphological indices, cerebral blood perfusion values and neuropsychological test results.

**Results:**

Compared with control group, UIA patients exhibited increased gray matter volume in the right anterior orbitofrontal cortex and decreased gray matter volume in the left thalamus pulvinar and hippocampus. Furthermore, the fractal dimension was lower in the right postcentral gyrus and entorhinal cortex. The cerebral perfusion values in the abnormal brain regions demonstrated a downward trend, which was associated with a reduction in gray matter volume in the left thalamus pulvinar and hippocampus, elevated anxiety levels and impaired executive function.

**Conclusion:**

UIA patients are prone to cognitive impairment and emotional dysregulation, which are accompanied by subtle changes in local gray matter volume and decreases in fractal dimension and cerebral blood flow. These findings provide new insights into the potential mechanisms underlying the cognitive impairment observed in UIA patients.

## 1 Introduction

An intracranial aneurysm is an abnormal bulge formed by the gradual expansion of intracranial arterial vessels under a hemodynamic load due to local vascular wall damage caused by congenital or acquired factors (1). With the popularization of imaging, the detection of unruptured intracranial aneurysms (UIAs) has increased substantially, with a prevalence of 3.2% among the general population (2). In the past, many neuropsychological studies have confirmed that patients with UIA are prone to emotional disorders and cognitive impairment, including impaired language, memory, visuospatial ability or cognitive decline (3).

Existing studies of UIAs have preliminarily explored the internal mechanism of the cognitive impairment observed in patients. Abnormalities in brain structure might be the neural correlates underlying these functional alterations (4, 5). In addition, the occurrence, development and rupture of aneurysms are closely related to hemodynamics. The cerebral hemodynamics of aneurysms are very complicated, but the accurate evaluation of resting cerebral perfusion is considered a marker for predicting changes in brain function because it is closely related to neuronal activity through neurovascular coupling (6). Increasing evidence suggests that even minor impairments in cerebral blood flow (CBF) regulation can have major effects on cognitive function (7).

In our study, we combined brain morphological analysis with perfusion imaging and conducted correlation analysis with neuropsychological test results to further explore the mechanism of cognitive impairment in UIA patients.

## 2 Methods

### 2.1 Participants

The inclusion criteria for the UIA group were as follows: (1) age 18–60 years; (2) follow-up observation selected after diagnosis by examination; and (3) absence of claustrophobia and other conditions contraindicated for 3.0 T MRI. The exclusion criteria were as follows: (1) arteriovenous malformations, epilepsy or other neurological or psychiatric diseases; (2) hypertension, diabetes, cancer or other chronic diseases; and (3) stimulating events such as recent surgical treatment or major family changes. All of the healthy controls (HCs) were in good health, and the exclusion criteria were similar to those of the UIA group. Finally, a total of 49 right-handed patients with UIAs and 50 right-handed HCs were recruited. All the subjects signed an informed consent form, and the study was approved by the Research Ethics Committee of Xinqiao Hospital (Chongqing, China).

### 2.2 Clinical scales and neuropsychological testing

Subjects completed the Perceived Stress Scale (PSS), a commonly used measure of stress. We also investigated the anxiety and depression levels of the subjects using the Self-Depression Scale (SDS) and Self-Anxiety Scale (SAS), which are widely used in the clinic and have good stability. Furthermore, a series of computerized cognitive tasks in the E-prime 2.0 program, including the two-back working memory task, the attention network test and the Stroop color-word test, were employed to assess working memory, attention and executive function (8).

### 2.3 MRI scanning

Arterial spin labeling magnetic resonance imaging (ASL-MRI) is a non-invasive MRI technique that enables the measurement of cerebral perfusion and provides more quantitative information. A 3.0 T Philips MRI system was employed to obtain T1-weighted high-resolution structural images with the following parameters: repetition time (TR) = 5.9 ms, echo time (TE) = 2.7 ms, flip angle (FA) = 15°, slices = 340, slice thickness = 0.5 mm, and gap = 0. Finally, ASL-MRI images were acquired using a pseudo-continuous ASL sequence (acquisition time = 430 s; post-labeling delay = 1800 ms; TR/TE = 4219/12 ms; FA = 90°; slices = 40; slice thickness = 3 mm, no gap; matrix = 80 × 80).

### 2.4 Image preprocessing

Preprocessing of the structural MRI data was performed using the Computational Anatomy Toolbox 12 (CAT12), an extension of Statistical Parameter Mapping 12 software (SPM12, https://www.fil.ion.ucl.ac.uk/spm). The toolbox covers diverse morphometric methods, such as voxel-based morphometry (VBM), surface-based morphometry (SBM) and region of interest (ROI)-based morphometry. VBM processing mainly involves tissue segmentation, bias correction and spatial normalization. The spatially adaptive nonlocal means denoising filter was employed during the segmentation process, followed by SPM’s standard unified segmentation (9). The output of this process aided in the optimization of CAT’s subsequent automatic segmentation. Spatial normalization registered the individual tissue segments to standardized templates in Montreal Neurological Institute (MNI) space. The modulated gray matter images were smoothed using an 8 mm full-width at half-maximum (FWHM) Gaussian kernel to facilitate statistical analysis. After data quality control, total intracranial volume (TIV) values were extracted as covariates for further analyses.

On the basis of the VBM processing, we performed SBM processing using the default recommended pipeline in CAT12, which included topological correction, spherical mapping, and spherical alignment (10). A 15 mm FWHM Gaussian kernel was used to smooth the resampled cortical thickness, and a 20 mm FWHM Gaussian kernel was used to obtain the gyrification index, sulcal depth and fractal dimension maps for each subject.

For the generation of ASL images, absolute quantified regional CBF maps were first generated by an embedded software tool in the 3.0 T Philips MRI scanner. SPM12 was subsequently employed to coregister the T1 images with the respective CBF maps and segment them into gray matter, white matter and cerebrospinal fluid. Each CBF map was then transformed into standard MNI space with 3 mm isotropic voxels. Finally, the transformed CBF maps were spatially smoothed using a 6 mm FWHM Gaussian kernel (11).

### 2.5 Statistical analysis

A statistical analysis of the demographic information and clinical neuropsychological test results was conducted using Statistical Product and Service Solutions 26 software (SPSS26). Pearson’s chi-square test and two-sample *t-tests* were used to compare categorical and continuous variables, respectively. If the variables were not normally distributed, the Mann–Whitney rank test was used. All *p*-values were set at <0.05, which was considered the threshold for statistical significance.

Before the ROI-based morphometry analysis, CAT12 divided the brain into different regions according to the Automated Anatomical Labeling atlas in the previous segmentation steps. A two-sample *t-test* was subsequently employed to examine regional differences in gray matter volume (GMV) in CAT12, with sex, age, education, and TIV serving as covariates. To perform the SBM analysis, ROI-based surface values were extracted using the embedded tool in CAT12. The subsequent stages of the process were analogous to those used in the VBM analysis, including the design of the basic model, estimation of the surface model, and ROI analysis. Importantly, TIV was not included as a covariate in the SBM analysis in this study. Finally, the differential brain regions obtained from the above analyses were employed to extract the corresponding CBF values for correlation analyses.

## 3 Results

### 3.1 Demographic, clinical, and neuropsychological results

Table 1 presents the demographic information, clinical data and neuropsychological test results. There were no significant differences in age, sex, years of education or smoking history between the 49 UIA patients and the 50 HCs. A total of 54 intracranial aneurysms were detected in 49 patients diagnosed with UIA. Predominantly, these aneurysms were small in size (less than 5 mm) and predominantly situated in the C6/7 segment of the internal carotid artery, accounting for 76% of the cases. The observed distribution of aneurysms corroborates the findings from a previous study (12). Furthermore, our analysis revealed no significant difference in the prevalence of UIAs between the left and right cerebral hemispheres. The subjects in the UIA group exhibited higher levels of anxiety and depression than those in the HC group. Compared with the HCs, UIA patients demonstrated longer alerting, orienting, and executive effect times on the attention network test. Furthermore, the UIA group demonstrated inferior performance to the HC group on the two-back working memory test, with a lower accuracy rating (ACC) and longer reaction time (RT). Although the RT on the executive function test was longer in the UIA group, there was no significant difference in the overall ACC between the two groups.

**Table 1:** Demographic, clinical characteristics, emotion and cognitive performance data.

Characteristic	UIA group	HC group	*p*-value
Sample (n)	49	50	
Age (years)	45.71 ± 8.52	42.82 ± 8.96	0.103^a^
Sex (male/female)	14 / 35	22 / 28	0.111^b^
Education (years)	11.31 ± 4.02	10.96 ± 3.19	0.637^a^
Smoking history (+/−)	8 / 41	13 / 37	0.239^b^
Disease duration (months)	24.18 ± 26.17		
Multiple aneurysms	5 (10.2%)		
Aneurysms size (mm)	4.30 ± 1.38		
Location			
ACoA	5 (9.26%)		
ACA (R)	1 (1.85%)		
MCA (R/L)	3 (5.56%)/1 (1.85%)		
PCoA	2 (3.70%)		
PCA (L)	1 (1.85%)		
C6 (R/L)	12 (22.22%)/17 (31.48%)		
C7 (R/L)	5 (9.26%)/7 (12.96%)		
Scales			
PSS	38.67 ± 8.01	29.00 ± 6.96	0.000^a^
SAS	49.03 ± 11.02	35.40 ± 7.76	0.000^a^
SDS	51.85 ± 9.82	40.56 ± 9.87	0.000^a^
Attention			
ANT alerting effect (ms)	99.24.32 ± 48.14	74.69 ± 42.32	0.008^a^
ANT orienting effect (ms)	32.75 ± 43.48	15.21 ± 32.15	0.024^a^
ANT executive effect (ms)	122.04 ± 64.65	95.51 ± 52.21	0.027^a^
Executive function			
ACC of stroop (%)	79.0 (60.0–90.0)	82.0 (70.0–93.0)	0.231^c^
Stroop RT (ms)	941.94 ± 197.43	770.23 ± 123.74	0.000^a^
Work memory			
ACC of two-back (%)	59.0 (50.5–71.5)	71.5 (59.5–79.3)	0.009^c^
Two-back RT (ms)	627.77 ± 141.72	541.42 ± 128.98	0.002^a^

PSS, perceived stress scale; SAS, self-rating anxiety scale; SDS, self-rating depression scale; ANT, attention network task; ACC, accuracy rating; RT, reaction time. ^a^Two-sample t-test; ^b^Chi-square test; ^c^Mann–Whitney rank test.

### 3.2 VBM, SBM and CBF analysis

**Figure 1:**
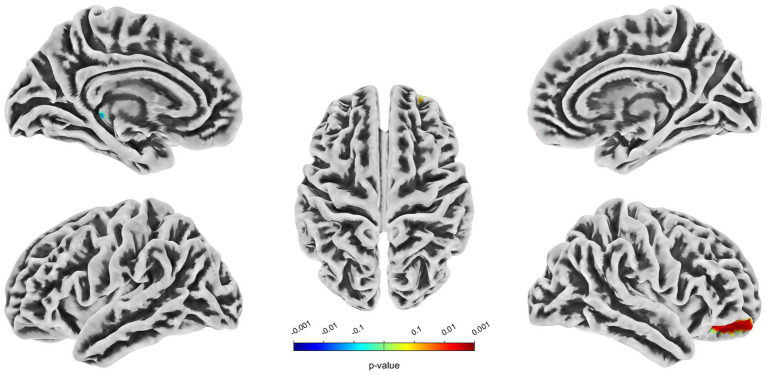
Significantly increased (red) and decreased (blue) gray matter volume in patients with UIA compared with HCs. The color bar represents the *p*-value of the two-sample *t*-test between the two groups.

**Figure 2:**
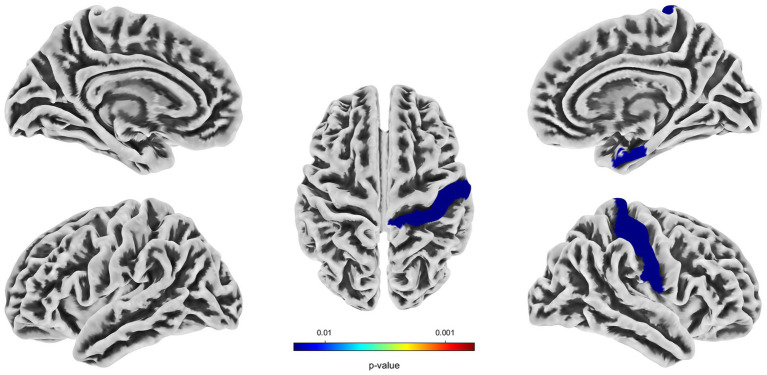
Significantly decreased (blue) fractal dimension in patients with UIA compared with HCs. The color bar represents the *p*-value of the two-sample *t*-test between the two groups.

Compared with the HC group, decreased GMV in the UIA group was observed in a small cluster, including the left thalamus pulvinar (tPUA.L) and left hippocampus (HIP.L). In addition, increased GMV was observed in the right anterior orbitofrontal cortex (OFCant.R) (*p* < 0.05, Holm–Bonferroni corrected) (Figure 1; Table 2). According to the SBM analysis, the fractal dimension in the UIA group was lower in the right postcentral gyrus (PoCG.R) and right entorhinal cortex (EC.R) (*p* < 0.05, Holm–Bonferroni corrected) (Figure 2; Table 3). In the analysis of SBM, the fractal dimension serves as a quantitative measure of the cerebral cortex’s intricacy. This metric adeptly captures the irregular yet self-similar morphological traits of structures, such as the cerebral cortex, and provides a quantitative framework to delineate the brain’s structural complexity (13). However, there was no significant difference between the two groups in cortical thickness, gyrification index, or sulcal depth. Finally, we used the abnormal brain regions acquired from the VBM and SBM analyses as ROIs and extracted the corresponding CBF values. The results presented in Table 4 indicate that the CBF values of the ROIs were lower in the UIA group.

**Table 2:** Regions of altered gray matter volume in UIA group in comparison with the HC group (Holm–Bonferroni corrected threshold of *p* < 0.05).

Cluster index	*p* value	*T* value	Peak MNI coordinate			Brain region	Voxels size
			*X*	*Y*	*Z*		
Cluster 1	0.03	−1.87	−18	−31	−5	tPUA.L HIP.L	163 51
Cluster 2	0.003	2.82	16	56	−24	OFCant.R	3,340

MNI, Montreal Neurological Institute; tPUA, thalamus pulvinar; HIP, hippocampus; OFCant, anterior orbitofrontal cortex.

**Table 3:** Regions of reduced fractal dimension in UIA group in comparison with the HC group (Holm–Bonferroni corrected threshold of *P* < 0.05).

Cluster index	*P* value	*T* value	Peak MNI coordinate			Brain region	Vertices size
			*X*	*Y*	*Z*		
Cluster 1	0.02	−2.52	40	−25	52	PoCG.R	1868
Cluster 2	0.01	−2.16	43	15	−32	EC.R	171

MNI, Montreal Neurological Institute; PoCG, postcentral gyrus; EC, entorhinal cortex.

**Table 4:** Comparison of CBF in ROI between UIA group and HC group.

Region of interest	Brain region	UIA group	HC group	*p*-value
ROI 1	tPUA.L / HIP.L	31.44 ± 6.99	35.57 ± 6.72	0.003^a^
ROI 2	OFCant.R	24.54 ± 5.26	27.61 ± 5.73	0.007^a^
ROI 3	PoCG.R	26.45 ± 6.88	29.64 ± 7.46	0.029^a^
ROI 4	EC.R	29.56 ± 6.53	33.94 ± 7.35	0.002^a^

tPUA, thalamus pulvinar; HIP, hippocampus; OFCant, anterior orbitofrontal cortex; PoCG, postcentral gyrus; EC, entorhinal cortex. ^a^Two-sample *t*-test.

### 3.3 Correlation analysis

**Figure 3:**
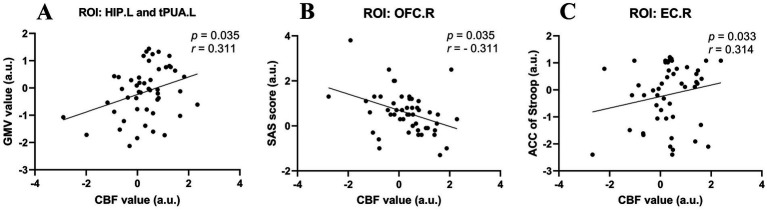
Scatter diagrams showing the partial correlation analysis among the abnormal GMV values, CBF values, and neuropsychological data in the UIA group in **(A–C)**. **(A)** The GMV of the tPUA.L and HIP.L was positively associated with the CBF values. **(B)** The CBF values of the OFCant.R was negatively associated with the SAS scores. **(C)** The CBF values of the EC.R was positively associated with the ACC on the Stroop test.

Considering that the age, sex and education of the subjects may have affected the results of the experiment, we performed partial correlation analysis among the abnormal GMV values, fractal dimension values, CBF values, clinical scale scores, and neuropsychological data in the UIA group. All the results with significant correlations are summarized in Figure 3. First, the GMV of the tPUA.L and HIP.L was positively associated with the corresponding CBF values (*p* = 0.035, *r* = 0.311). Second, there was a negative correlation between the CBF values of the OFCant.R and SAS scores (*p* = 0.035, *r* = − 0.311). Third, the CBF values of the EC.R showed a positive correlation with the ACC on the Stroop test (*p* = 0.033, *r* = 0.314). In a further step, we investigated the effect of disease duration on emotion, cognitive function, CBF and brain morphology by correlation analysis in the UIA group, but no positive results were found.

## 4 Discussion

To our knowledge, this is the first study to use brain structure and perfusion MRI to explore the mechanism of cognitive impairment in UIA patients. The current study demonstrated that the brain regions exhibiting structural abnormalities and regional cerebral hypoperfusion are closely associated with cognitive function and emotion.

Previous studies have reported that risk factors for UIA, such as smoking and hypertension, may cause the thalamus to be vulnerable to change (14, 15). Although no atrophy of the total thalamic or hippocampal volume was observed, decreased GMV was identified in a small cluster, including parts of the tPUA.L and HIP.L, in UIA patients. Some studies of brain morphology have shown similar results, with focal thalamic atrophy reported in patients with UIAs (5). A more profound comprehension of the role of the thalamus reveals its indispensable involvement in a multitude of brain functions, including consciousness, sleep, memory, sensation, and movement (16, 17). The thalamus also serves as a processing and distribution center, relaying and regulating information from the external and internal environment to the cerebral cortex (18, 19). Research has demonstrated that hippocampal atrophy is associated with short-term memory loss, which is commonly observed in the early stages of Alzheimer’s disease (AD) (20). However, hippocampal atrophy may not be specific to AD (21). As the principal region of the brain responsible for learning and memory, the hippocampus is intimately associated with memory, stress regulation and spatial navigation processes (22). We suggest that atrophy of local areas of the hippocampus and thalamus may account for impaired memory and attention functions. Furthermore, the GMV of the tPUA.L and HIP.L exhibited a positive correlation with the corresponding CBF values. Given that the hippocampus is one of the cerebral regions most susceptible to ischemic damage, any cause of local microenvironmental blood supply or metabolic abnormalities in the hippocampus can result in hippocampal atrophy (23). It is further postulated that diminished local blood perfusion may be a pivotal factor contributing to atrophy.

The present study revealed that the UIA group exhibited worse emotional states, including elevated stress, anxiety, and depression levels, than did the HC group. In addition, UIA patients exhibited lower CBF values in the OFCant.R than HCs did, and the decreased CBF values were negatively correlated with SAS scores. Some studies have indicated that the OFC is involved in executive function, decision-making, and the neural basis of human emotions, including pleasure, embarrassment, anger, and sadness (24, 25). In short, the OFC uses our emotional responses to guide our behavior and control our emotions in different social situations. These results suggested that the anxiety observed in UIA patients may be related to a reduction in CBF. Furthermore, UIA patients exhibited increased GMV in the OFCant.R compared with HCs. It’s different from what we usually think of as hypoperfusion causing brain atrophy, some studies have found atrophy of gray matter in the right OFC with high perfusion (26). Other studies have shown that brain function is tightly coupled with CBF, suggesting that regional CBF is a quantitative index of metabolic activity within specific networks (27). Those findings suggest ASL MRI may be sensitive to functional changes not readily apparent in structural MRI. We suspect that hypoperfusion in the OFC causes abnormal brain function, and the observed increase in GMV may reflect an increase in compensatory “hypertrophic” regions in the presence of abnormal alterations in brain function (28). However, we must recognize that this explanation remains highly speculative. Future research must confirm these findings and follow up further on their mechanistic basis.

In recent years, studies have confirmed the role of the EC in spatial learning and memory (29). It is the principal pathway between the hippocampus and the neocortex, and its malfunction can result in cognitive impairment and dementia (30). Some studies have also indicated a correlation between reduced cerebral perfusion or GMV in the EC and cognitive impairment (31, 32). Despite the absence of statistically significant atrophy of the EC in our study, the results of SBM analyses indicated a reduction in fractal dimension values of the EC.R and PoCG.R when compared to the healthy group, accompanied by a decrease in cerebral blood perfusion. In addition, correlation analysis revealed that reduced CBF values were associated with impaired executive function in UIA patients. Our findings indicate that subtle alterations in local CBF and brain structure may occur in the early stages of cognitive impairment. It is postulated that SBM analyses may be more sensitive than VBM analyses in detecting subtle structural changes in the brain.

Previous studies have also identified numerous connections between the PoCG and other brain regions, including the cerebellum, amygdala, limbic system, and parietal lobe (33). Owing to its specific location, structure, and function, the PoCG is capable of performing a multitude of functions. Some studies using structural and resting-state functional MRI have revealed a correlation between abnormal changes in the PoCG and anxiety and depressive disorders (34, 35). A further study demonstrated that reduced cortical thickness in the PoCG was associated with cognitive impairment (36), whereas increased surface area of the PoCG was associated with better executive function (37). The PoCG is not only an important structure in the somatosensory network but also associated with emotion disorders and participates in the regulation of cognitive function. Our results demonstrated that patients exhibited diminished fractal dimension values and CBF in the PoCG.R. Despite the absence of a significant correlation among the abnormal fractal dimension values, CBF values, clinical scale scores, and neuropsychological results in the UIA group, it is reasonable to hypothesize that the reduction in cortical complexity and cerebral hypoperfusion as the disease progresses may contribute to the patient’s cognitive impairment and emotional disorders.

Finally, we acknowledge that our study has several limitations. Because of the small sample size, the study is not able to further group by aneurysm location to explore the effect of different aneurysm locations on brain structure and cerebral blood perfusion. Moreover, our study is a cross-sectional design and it cannot observe these abnormal alterations over time. Future studies should adopt a long-term cohort design with an increased sample size to ameliorate these limitations.

## 5 Conclusion

In conclusion, UIA patients are prone to cognitive impairment and emotional dysregulation, accompanied by subtle changes in local GMV and a decrease in fractal dimension values and CBF. Consequently, our findings may offer new insights into the potential mechanisms underlying the cognitive impairment observed in UIA patients. Furthermore, these findings may provide clinicians with additional references for early intervention in patients with UIAs.

## Data Availability

The raw data supporting the conclusions of this article will be made available by the authors, without undue reservation.
